# A new species of trogloplacine crab of the genus *Australocarcinus* Davie, 1988 from a freshwater stream in Mahé, Seychelles (Crustacea, Brachyura, Chasmocarcinidae)

**DOI:** 10.3897/zookeys.738.23708

**Published:** 2018-02-19

**Authors:** Peter K. L. Ng, Savel R. Daniels

**Affiliations:** 1 Lee Kong Chian Natural History Museum, 2 Conservatory Drive, National University of Singapore, Singapore 117377, Republic of Singapore; 2 Department of Botany and Zoology, University of Stellenbosch, Private Bag X 1, Matieland, 7602, South Africa

**Keywords:** Chasmocarcinidae, freshwater, Indian Ocean, new species, Trogloplacinae, taxonomy

## Abstract

A new species of freshwater chasmocarcinid crab, *Australocarcinus insperatus* **sp. n.**, is described from the Seychelles Islands in the Indian Ocean. This is the first record of the genus and the subfamily Trogloplacinae Guinot, 1986, from the Indian Ocean, with all other members previously recorded from Australia, New Britain, New Caledonia, and Palau in the Pacific Ocean. The disjunct distribution of *Australocarcinus* is unexpected considering all trogoplacines are believed to practice direct development, lacking free-swimming larval stages. The new species is morphologically most similar to *A. riparius* Davie, 1988, from Queensland, Australia, but can be distinguished from its three congeners on the basis of the structures of its carapace, ambulatory legs and male first gonopod.

## Introduction

The Chasmocarcinidae Serène, 1964, is a predominantly marine family, with most of the species occurring in subtidal habitats, and some reaching depths of over 2000 metres (Ng and Castro 2016). One subfamily, the Trogloplacinae Guinot, 1986, however, occurs exclusively in freshwater habitats, sometimes several kilometres from the sea as well as in aquatic inland limestone caves (Davie and Guinot 1996; Ng and Castro 2016). Trogloplacines are also unusual in practising direct development, lacking planktotrophic larvae (Davie and Guinot 1996). Only two genera of Trogloplacinae are known, the monotypic *Trogloplax* Guinot, 1986 (which lives in caves in New Britain, southwestern Pacific), and *Australocarcinus* Davie, 1988 (with three epigeal species from northeastern Australia, New Caledonia and Palau). The first *Australocarcinus* species is reported here from the Indian Ocean, *A. insperatus* sp. n., from southern Mahé in the Seychelles archipelago.

## Materials and methods

Material examined is deposited in the Zoological Reference Collection (**ZRC**) of the Lee Kong Chian Natural History Museum, National University of Singapore. Measurements provided (in millimetres) are of the carapace width and length, respectively. The terminology used follows that in Ng and Castro (2016) and Davie et al. (2015). The following abbreviations are used: **G1** male first pleopod; **G2** male second pleopod.

## Systematics

### Family Chasmocarcinidae Serène, 1964

#### Subfamily Trogloplacinae Guinot, 1986

##### Genus Australocarcinus

Taxon classificationAnimaliaDecapodaChasmocarcinidae

Davie, 1988

###### Type species.

*Australocarcinus riparius* Davie, 1988, by original designation.

##### Australocarcinus insperatus sp. n.

Taxon classificationAnimaliaDecapodaChasmocarcinidae

http://zoobank.org/909E98B1-A957-44F0-BD2E-2C665E67CFB1

Figs 1 , 2 , 3

###### Material examined.

Holotype: male (10.7 × 8.6 mm) (ZRC 2017.1072), in shallow stream, ca. 800 m from sea, about 2 km south-southeast of international airport, 4°41'32.42"S, 55°31'2.90"E, Mahé, Seychelles, coll. SR Daniels, May 2010. Paratypes: 1 male (8.5 × 7.2 mm), 1 female (9.5 × 7.8 mm) (ZRC 2017.1073), same data as holotype.

###### Diagnosis.

**Figure 1.**
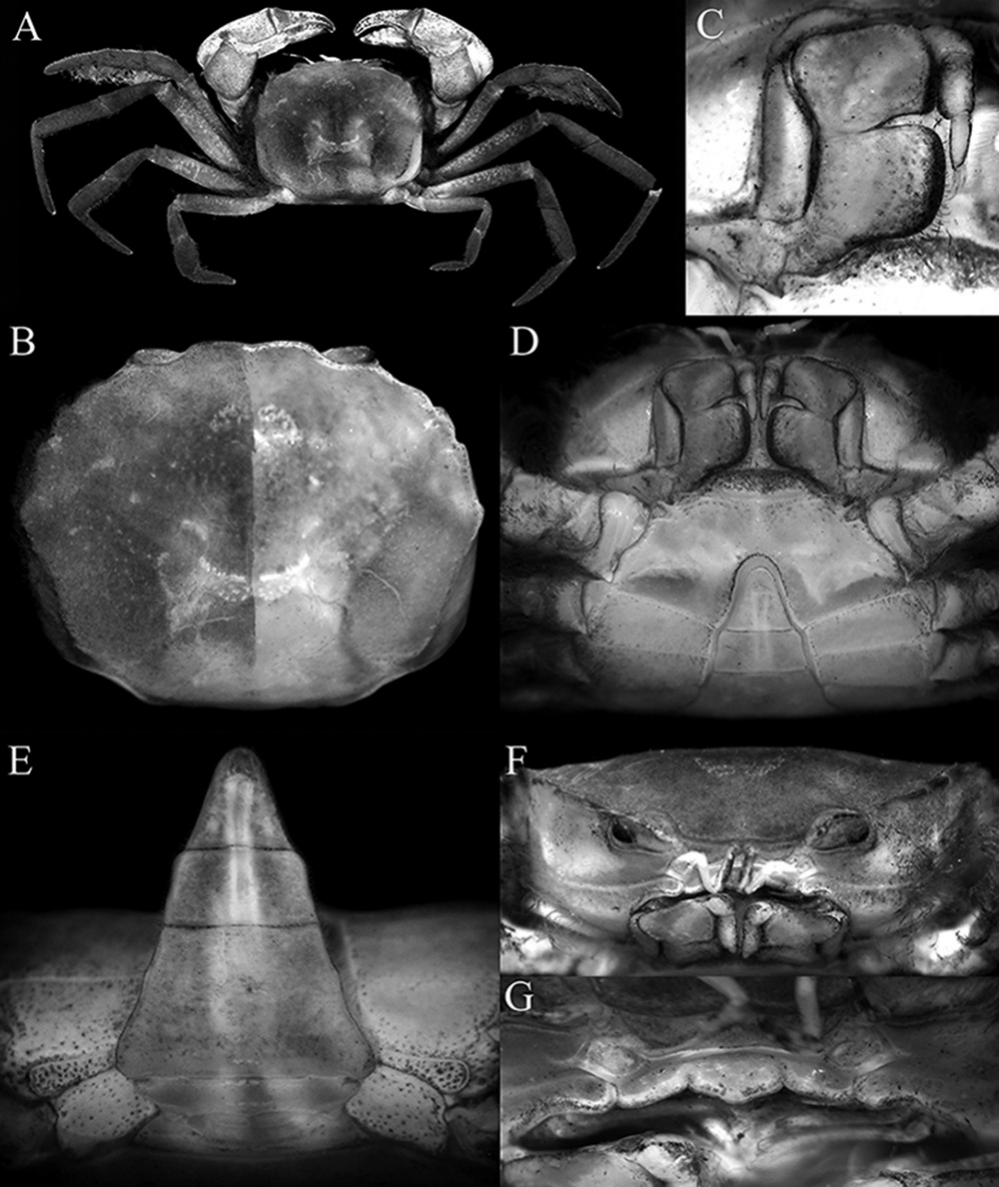
*Australocarcinus insperatus* sp. n., holotype male (10.7 × 8.6 mm) (ZRC 2017.1072), Seychelles. **A** overall dorsal habitus **B** dorsal view of carapace (right side denuded) **C** right third maxilliped (denuded) **D** anterior thoracic sternum and pleon **E** posterior thoracic sternum and pleon **F** frontal view of cephalothorax **G** posterior margin of epistome.

**Figure 2.**
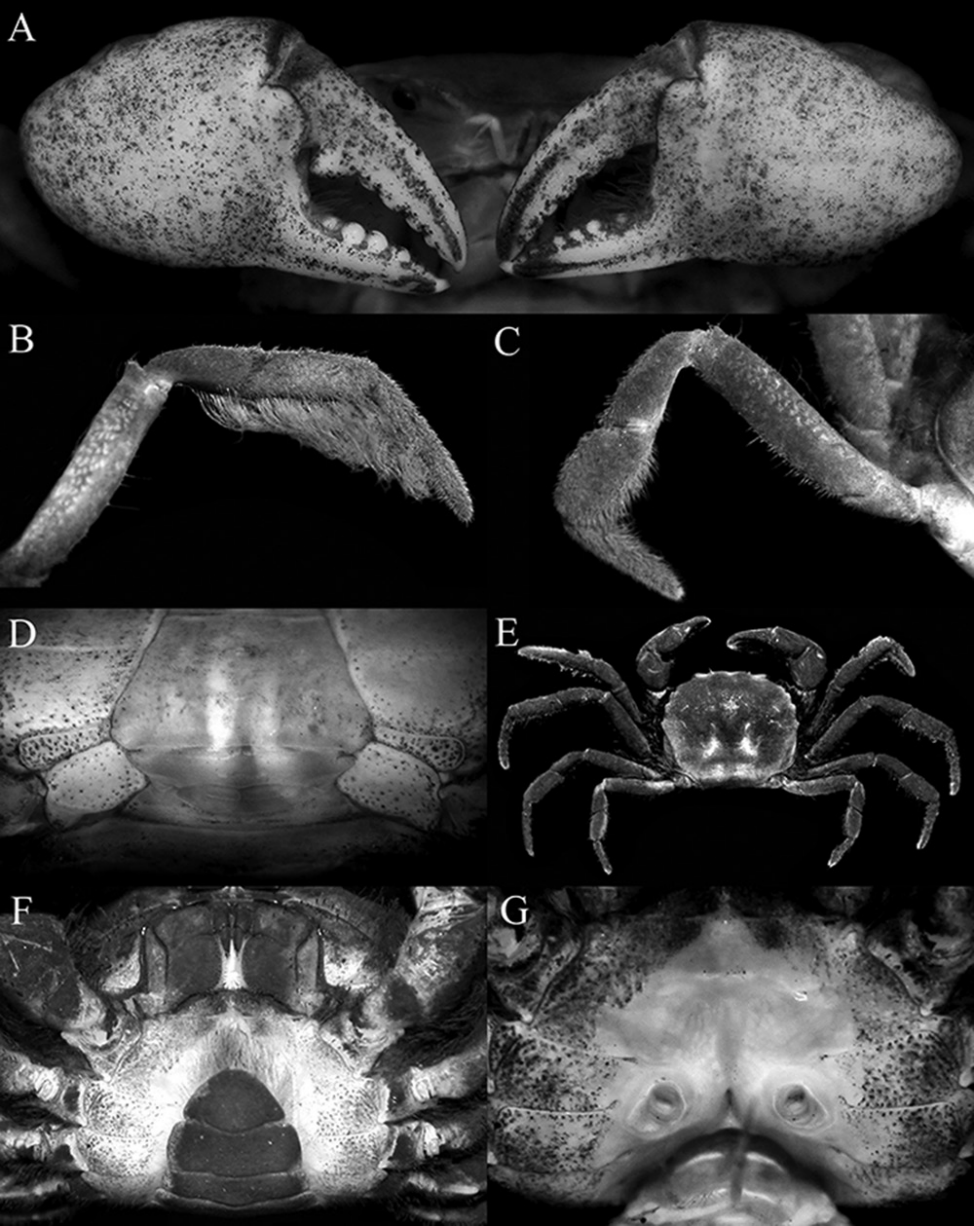
*Australocarcinus insperatus* sp. n. **A**–**D** holotype male (10.7 × 8.6 mm) (ZRC 2017.1072), Seychelles **E**–**G** paratype female (9.5 × 7.8 mm) (ZRC 2017.1073), Seychelles. **A** outer surfaces of chelae **B** right first ambulatory leg showing setose posterior margin on propodus and dactylus **C** left fourth ambulatory leg **D** posterior thoracic sternum showing supplementary plate **E** female overall dorsal habitus **F** female posterior thoracic sternum and pleon **G** female sterno-pleonal cavity showing vulvae.

**Figure 3.**
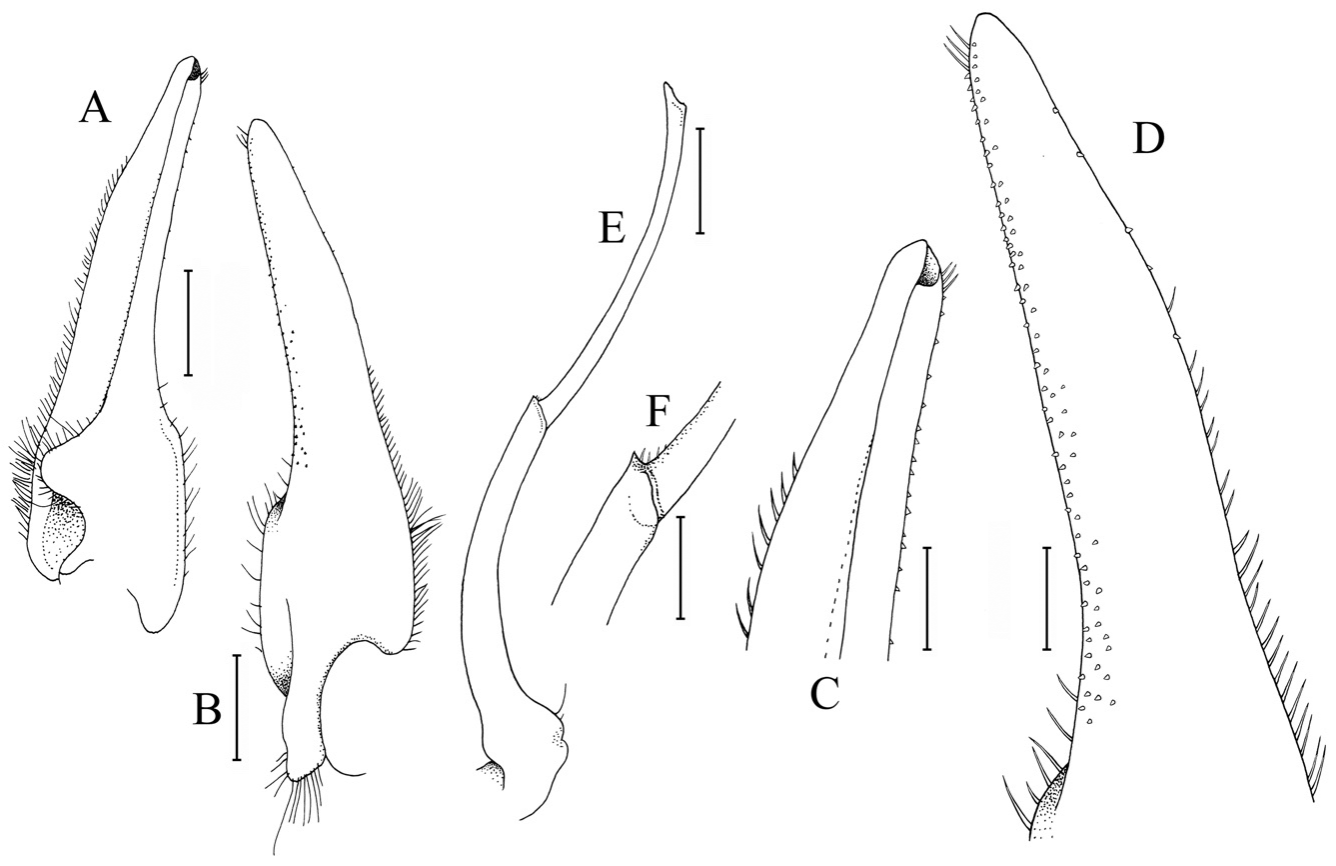
*Australocarcinus insperatus* sp. n., left G1 and G2; holotype male (10.7 × 8.6 mm) (ZRC 2017.1072), Seychelles. **A** ventral view **B** ventral view **C** distal part (ventral view) **D** distal part (dorsal view) **E** ventral view **F** area between basal and distal segments. Scale bars 0.50 mm (**A, B, E**); 0.25 mm (**C, D, F**).

Carapace subquadrate, front weakly bilobed, with shallow median concavity (Fig. 1A, B); dorsal surface gently convex (Fig. 1F); dorsal surfaces and margins covered with short uneven tomentum (Fig. 1A, B); anterolateral margins arcuate, with four low teeth: first widest with gently sinuous margin, second lobiform, third wide, fourth (at junction of antero- and posterolateral margins) dentate, directed laterally, protruding beyond margin (Fig. 1B). Posterolateral margin converging towards gently convex posterior carapace margin (Fig. 1B). Epistome compressed, posterior margin with distinct triangular median lobe with median fissure, lateral margins gently sinuous (Fig. 1G). Eye peduncle completely filling orbit, relatively short, mobile; cornea distinct, pigmented (Fig. 1B, F). Third maxillipeds leaving gap when closed; merus quadrate, anteroexternal angle auriculiform; ischium quadrate, slightly longer than merus with very shallow median sulcus (Fig. 1C, D). Chelipeds subequal, relatively stouter in males (Figs 1A, 2E); cutting margins of both chelae with distinct teeth in both sexes, base of fingers with tuft of stiff setae; proximal part of dactylus of right chela with large, triangular tooth directed towards palm (Fig. 2A); ventral surface of cheliped merus with tubercles. Ambulatory legs moderately short; meri unarmed but setose to varying degrees; P2 carpus, propodus and dactylus with very long coarse setae which obscures margins (Figs 1A, 2B); P3–P5 propodus and dactylus setose but setae shorter than on P5 (Fig. 2C); P5 dactylus straight (Fig. 2C). Thoracic sternites 1, 2 fused, broadly triangular, short; separated from sternite 3 by sinuous groove; sternites 3, 4 fused, relatively broad (Fig. 1D). Male pleon with lateral margins of somite 6 and fused somites 3‒5 gently sinuous; telson slightly longer than broad (Fig. 1D, E). Sterno-pleonal cavity of male deep, press-button for pleonal holding small, short tubercle posterior to thoracic sternal suture 4/5 near edge of sterno-pleonal cavity. Male thoracic sternite 8 short, rectangular; supplementary plate narrow, wider along outer part (Figs 1E, 2D). G1 stout; basal part truncate; distal part cylindrical, with rounded tip, covered with short spinules (Fig. 3A–D). G2 prominently longer than G1, basal segment curved; distal segment slightly longer than basal segment, apex cup-like (Fig. 3E, F). Somites of female pleon with slightly convex lateral margins; telson wider than long (Fig. 2F). Sterno-pleonal cavity of female moderately deep, with large vulvae distinctly separated from each other, covering most of thoracic sternite 5, ovate, with low raised lip on outer margin, opening slit-like (Fig. 2G).

###### Etymology.

From the Latin “*insperatus*” for “unforeseen”, alluding to the unexpected discovery of a species of *Australocarcinus* in the western Indian Ocean.

###### Remarks.

Davie (1988) originally established *Australocarcinus* for one freshwater species from northern Queensland in Australia, *A. riparius* Davie, 1988. Davie & Guinot (1996) subsequently described two more species, *A. kanaka* Davie & Guinot, 1996, and *A. palauensis* Davie & Guinot, 1996, from New Caledonia and Palau, respectively. Davie & Guinot (1996) showed that *Australocarcinus* was in the same subfamily as the more apomorphic cavernicolous species *Trogloplax joliveti* Guinot, 1986, from New Britain; that it belonged to the family Chasmocarcinidae; and provided evidence that their larval development was truncated with the eggs hatching directly into juvenile crabs or megalopas.

**Figure 4.**
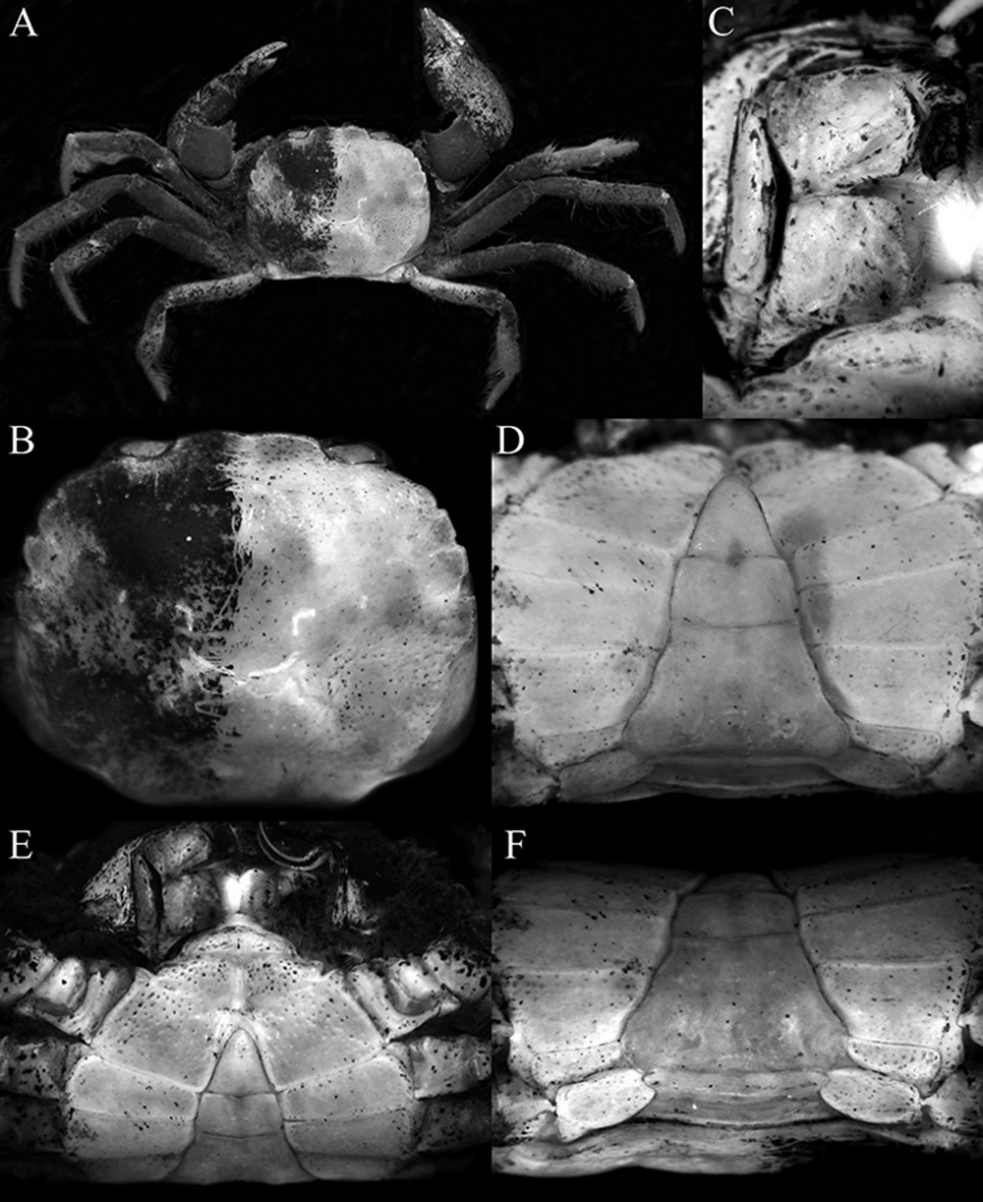
*Australocarcinus riparius*, male (8.8 × 10.2 mm) (ZRC 2006.167), Australia. **A** overall dorsal habitus **B** dorsal view of carapace (right side denuded) **C** right third maxilliped (denuded) **D** posterior thoracic sternum and pleon **E** anterior thoracic sternum and pleon **F** posterior thoracic sternum showing supplementary plate.

*Australocarcinus insperatus* sp. n., is morphologically most similar to *A. riparius* Davie, 1988, in the anterolateral margin possessing four low teeth, the anteroexternal angle of the merus of the third maxilliped is clearly auriculiform and the male telson is relatively longer. *Australocarcinus insperatus* sp. n., however, can easily be separated by possessing a more sub-hexagonal carapace (Fig. 1B) (vs. carapace more subquadrate in *A. riparius*, Fig. 4B); a distinctly convergent posterolateral margin (Fig. 1B) (vs. posterolateral margins subparallel in *A. riparius*, Fig. 4B); the last anterolateral tooth is triangular and protrudes laterally beyond the carapace margin (Fig. 1B) (vs. last tooth truncate and not extending beyond carapace margin in *A. riparius*, Fig. 4B); the ischium of third maxilliped is wider than long (Fig. 1C) (vs. ischium longer than wide in *A. riparius*, Fig. 4C); the ambulatory merus is more elongate and slender (Figs 1A, 2B, C, E) (vs. meri proportionately shorter in *A. riparius*, Fig. 4A); and the G1 is relatively more slender (Fig. 3A–D) (vs. G1 stouter in *A. riparius*, cf. Ng and Castro 2016: fig. 98A).

All three specimens of *A. insperatus* sp. n. have a distinct cutting or peeling tooth at the base of the dactylus of the right chela (Fig. 2A), a character which Ng and Tan (1984, 1985) have suggested is used to specially feed on gastropod snails. As most gastropod snails have dextral coiling (opening on the right side when viewed frontally), Ng and Tan (1984, 1985) observed that crabs with the enlarged basal dactylar tooth always have this structure on the right chela to make peeling of the shell more efficient. The other three species of *Australocarcinus* also have this tooth on the right chela (see Ng and Castro 2016: figs 95A, C, E) and on both sexes. This suggests that one of the main food items of *Australocarcinus* are freshwater gastropods.

The discovery of *A. insperatus* sp. n. is surprising as all the members of the Trogloplacinae have been previously found in Australasian and Palau waters. Davie (1988) found juvenile crabs under the pleon of a female *A. riparius*, with ovigerous specimens possessing some 70 large eggs. Davie and Guinot (1996) found megalopa under a female pleon of *A. kanaka*, suggesting that the development was direct, like those in primary freshwater crabs like Potamidae, Potamonautidae and Gecarcinucidae (and some Sesarmidae). All trogloplacines also have large vulvae (Ng and Castro 2016: figs 99B, D, F, H), suggesting the eggs of the other two species, *A. palauensis* and *Trogloplax joliveti* also have large eggs and do not have free-swimming larvae. The vulvae of *A. insperatus* sp. n. are also large (Fig. 2G). If all trogloplacines have abbreviated (or at least a semi-abbreviated) development and there are no free-swimming larvae, how did they disperse so widely? Despite hypotheses that primary freshwater crabs may have dispersed through Gondwanic connections (Ng et al. 1995), the available evidence is that they are not old enough to have done so (see Daniels et al. 2006; Cumberlidge et al. 2008; Klaus et al. 2009; Cumberlidge and Ng 2009; Daniels 2011; Cumberlidge and Daniels 2014); and as such, the disjunct distribution of *A. insperatus* sp. n. begs further studies. A complete molecular phylogeny of the Chasmocarcinidae is now being undertaken by L. M. Tsang (Chinese University of Hong Kong) and the results should throw some light on this matter in the future.

###### Biology.

The freshwater stream where the specimens were collected was shallow, the water flowing over a sandy bottom, with scattered rocks and construction rubble from past development works in the area. The crabs attempted to bury into the soft sand when disturbed.

#### Amended key to species of *Australocarcinus*

**Table:** 

1	Anterolateral margin entire, without visible lobes or teeth [Palau]	***A. palauensis***
–	Anterolateral margin distinctly dentate	**2**
2	Anterolateral margin with 2 low, blunt lobes; anterolateral margin of merus of third maxilliped rounded, not auriculiform; male telson relatively short [New Caledonia]	***A. kanaka***
–	Carapace anterolateral margin with 4 prominent but low teeth; anterolateral margin of merus of third maxilliped expanded, auriculiform; male telson relatively long	**3**
3	Carapace subquadrate, posterolateral margin subparallel; last anterolateral tooth truncate, not protruding laterally beyond carapace margin; ischium of third maxilliped longer than wide; ambulatory merus relatively shorter, stouter; G1 stout [Queensland, Australia]	***A. riparius***
–	Carapace subhexagonal, posterolateral margin gently converging; last anterolateral tooth triangular, distinctly protruding laterally beyond carapace margin; ischium of third maxilliped wider than long; ambulatory merus prominently elongate, slender; G1 relatively more slender [Seychelles]	***A. insperatus* sp. n.**

## Supplementary Material

XML Treatment for Australocarcinus

XML Treatment for Australocarcinus insperatus
